# Food fear, quick satiety and vomiting in a 16 years old girl: It’s bulimia, or maybe not…? A case report of Wilkie’s syndrome (superior mesenteric artery syndrome)

**DOI:** 10.1016/j.ijscr.2019.10.038

**Published:** 2019-11-06

**Authors:** Giovanni Frongia, Jens-Peter Schenk, Anja Schaible, Peter Sauer, Arianeb Mehrabi, Patrick Günther

**Affiliations:** a Division of Pediatric Surgery, Department of General, Visceral and Transplantation Surgery, University Hospital of Heidelberg, Germany; b Division of Pediatric Radiology, Department of Diagnostic and Interventional Radiology, University Hospital of Heidelberg, Germany; c Interdisciplinary Endoscopy Center, Department of General, Visceral and Transplantation Surgery, University Hospital of Heidelberg, Germany; d Interdisciplinary Endoscopy Center, Department of Gastroenterology and Hepatology, University Hospital of Heidelberg, Germany; e Department of General, Visceral and Transplantation Surgery, University Hospital of Heidelberg, Germany

**Keywords:** Wilkie syndrome, Superior mesenteric artery syndrome, Duodenal stenosis, Vomiting, Duodeno-jejunostomy, Case report

## Abstract

**Introduction:**

Wilkie’s syndrome (WS), also known as superior mesenteric artery syndrome, is a rare clinical entity caused by compression of the horizontal (third) part of the duodenum between the superior mesenteric artery and the abdominal aorta leading to duodenal obstruction.

**Presentation of case:**

We report a case of a 16 years girl with long-term history of spontaneous vomiting and self-induced vomiting, also suffering from recurrent retrosternal pain, weight loss and thus reduced quality of life. Contrast intestinal series showed a large axial hernia which was laparoscopically reduced and treated by hiatoplasty and anterior hemifundoplication. After initial relief, recurrent postprandial nausea and vomiting reoccurred 4 weeks postoperatively. Abdominal MRI study revealed findings compatible with WS. After endoscopic exclusion of an anatomical duodenal lumen stenosis, she was successfully treated by duodeno-jejunostomy with a favorable outcome.

**Discussion:**

WS might be hidden behind presumably more evident diagnoses such as bulimia, significant axial hernia and gastro-esophageal reflux disease in patients with recurred vomiting, abdominal pain and weight loss.

**Conclusion:**

The rare clinical entity of a WS necessitates a targeted diagnostic evaluation and therapy. Clinical details, diagnostic studies and treatment are discussed here.

## Highlights

• Wilkie’s syndrome (WS) is a rare but impairing condition.
• WS is defined by an aorto-mesenteric artery angle <25° or an aorto-mesenteric distance of <8 mm.
• Conservative approaches aim at restoring the correct aorto-mesenteric artery angle.
• Surgical approaches aim at bypassing the functional duodenal obstruction.
• The overall WS outcome is good in 80–90% of cases.

## 1 Introduction

Wilkie’s syndrome (WS), also known as superior mesenteric artery syndrome, is a rare clinical entity. It predominantly affects women and the main manifestation age is between 10 and 39 years [1]. WS causes a compression of the horizontal part of the duodenum between the superior mesenteric artery and the abdominal aorta leading to duodenal obstruction. We present and discuss a case of WS in a young girl placing emphasis on targeted evaluation and surgical therapy managed at a university pediatric surgery center. This work has been reported in line with the SCARE [2].

## 2 Presentation of case

**Fig. 1:**
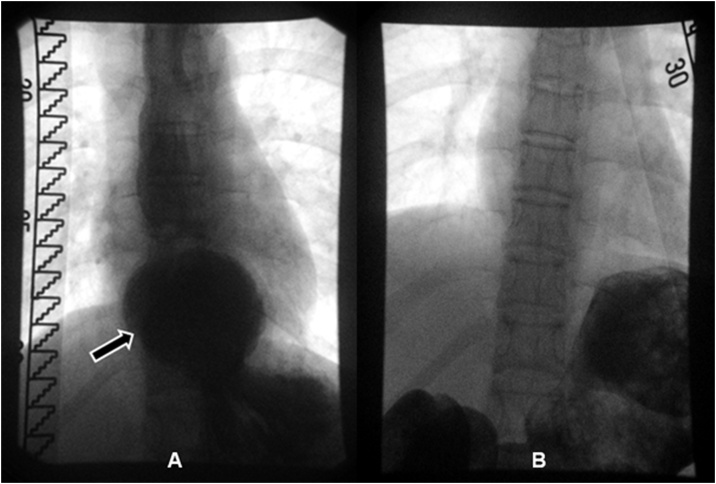
Upper gastrosintestinal contrast series (A) prior to laparoscopic surgery showing a large axial hernia with gastro-esophageal reflux (arrow) and (B) four weeks after successful laparoscopic hernia reduction, hiatoplasty and anterior hemifundoplication, showing successful hernia reduction and antireflux therapy.

**Fig. 2:**
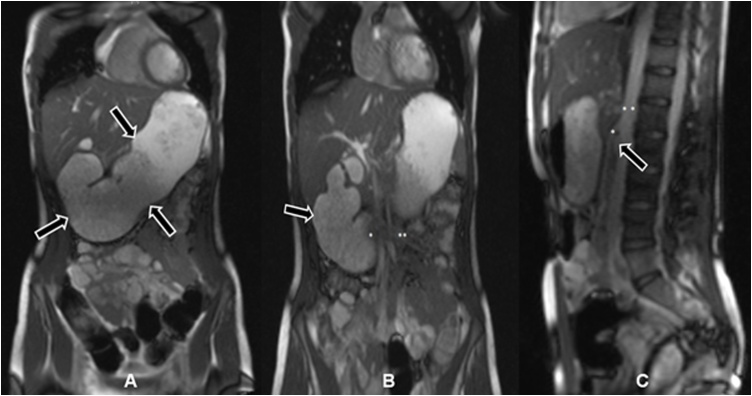
Abdominal MRI showing (A) a dilated stomach (arrows), (B) a dilated duodenum (arrow) with change in caliber from before (*) to after (**) under passing the mesenteric root. Further an duodenal obstruction (C) between the superior mesenteric artery (*) and the abdominal aorta (**) can be seen. This duodenal obstruction is caused by a aorto-mesenteric angle of 14°, which is compared to normal angle values of 40–55°. These findings are compatible with a Wilkie’s syndrome (superior mesenteric artery syndrome) diagnosis.

**Fig. 3:**
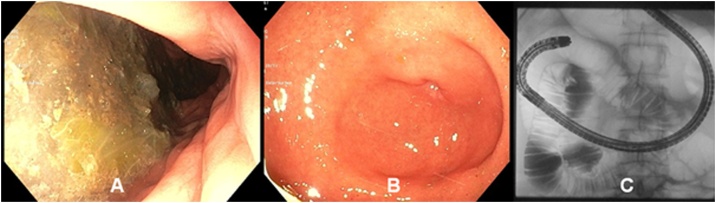
Endoscopy showing (A) significant gastric food leftovers despite oral food waiver for approximately 24 h, (B) exclusion of a tangible anatomical luminal duodenal stricture and (C) radiological and endoscopic proof of an inconspicuous complete intestinal into the proximal jejunal loops.

**Fig. 4:**
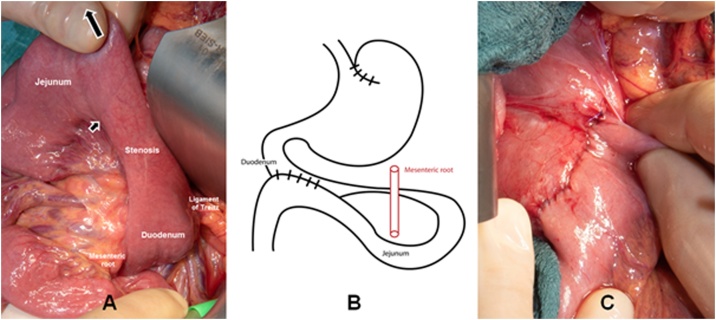
Intraoperative view showing (A) manually pulled out (large arrow) duodenum from under the mesenteric root presenting a change in caliber and a clear demarcation line towards the jejunum (small arrow); (B) graphic illustration of the bypass operation between duodenum an proximal jejunum to obviate the duodenal stenosis at the height of the mesenteric root; (C) side-to-side duodeno-jejunostomy with a continuous double-layer resorbable monofilament suture.

**Fig. 5:**
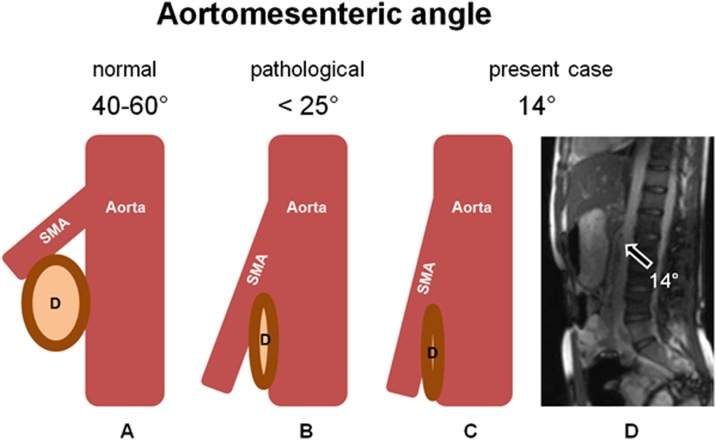
Graphic illustration of (A) the normal angle of 40–60° between the superior mesenteric artery (SMA) and the aorta allowing a regular luminal diameter of the duodenum (D), in contrast to (B) a pathological low angle of <25° in Wilkie’s syndrome resulting in duodenal compression, and (C) an angle of 14° in the here present case, resulting in a clinical relevant duodenal compression (arrow).

A 16-year-old girl presented to our consultation with complaints of long-term severe spontaneous vomiting and regurgitation during sports or mundane movements, such as leaning forward to tie shoes. Moreover she reports regular retrosternal pain, acid mouth taste along with a body weight loss (BMI 17.5). These symptoms led to food fear, quick satiety and a clearly reduced quality of life. Two years prior she underwent a phase of bulimia with self-induced vomiting accompanied by recurrent abdominal pain and weight loss (BMI 10.5). Psychotherapy and behavioral therapy helped overcome this phase successfully leading to weight gain (BMI 18.5) and renewed desire and pleasure to regularly eat. She was also under drug therapy with Mirtazapine for a depression. Physical examination was inconspicuous and laboratory values within normal limits. Esophageal contrast intestinal series showed a large axial hernia with gastro-esophageal reflux (Fig. 1A), compatible with an anatomical cause of gastro-esophageal-reflux-disease, and a normal gastric emptying. A laparoscopic axial hernia reduction, hiatoplasty and anterior hemifundoplication initially led to symptoms relief, but recurrent postprandial nausea and vomiting reoccurred 4 weeks postoperatively. The postoperative upper gastrointestinal contrast series were unsuspicious (Fig. 1B). The patient experienced subjectively reduction of symptoms under oral therapy with erythromycin, taking advantage of its propulsive side-effect with almost no significant antibiotic effect at low doses (3 mg/kg, 4 times a day), after unsatisfactory propulsive oral medication attempts with dimenhydrinate, and dietary changes with frequent and small food servings. Due to persisting symptoms for 5 months, despite these conservative treatment attempts, the patient underwent abdominal MRI study. This revealed a dilated stomach and duodenum, with a duodenal change in caliber underrunning the mesenteric root, compatible with a duodenal obstruction between the superior mesenteric artery and the abdominal aorta caused by a smaller angle between the two vessels of 14° (normal angle values 40–60° [3,4]) (Fig. 2). These findings were compatible with a Wilkie’s syndrome (WS) diagnosis. An upper gastrointestinal tract endoscopic study revealed significant gastric food leftovers despite oral food waiver for approximately 24 h, excluded a tangible anatomical luminal duodenal obstruction and provided a radiological and endoscopic proof of an inconspicuous intestinal passage into the proximal jejunal loops (Fig. 3). At this point indication for surgery was set, based on the patient restricted quality of life with difficulties to conduct a normal daily routine and the impossibility to enable a clinical satisfied state by conservative approaches over several months. The patient underwent laparotomy with confirmation of a functional duodenal obstruction and duodenal change in caliber underpassing the mesenterial root (Fig. 4). A standardized side-to-side duodeno-jejunostomy with a continuous double-layer resorbable monofilament suture (PDS 4-0, Ethicon, Norderstedt, Germany) was performed to bypass the duodenal obstruction (Fig. 4). The postoperative course was uneventful and the patient was discharged on the sixth postoperative day. No complications, adverse or unanticipated events occurred at short term. At 8 weeks follow-up the patient is free of significant symptoms, regained body weight (BMI 19.5) and reports a subjectively normal quality of life. Further follow-ups for clinical investigations are planned (Fig. 5).

## 3 Discussion

A WS was first described in 1842 [5] and in the 1920’s named after a David Wilkie, a Scottish surgeon [6,7]. About 450 original articles and reviews were published on this condition from 1950 to July 2019 on PubMed (www.ncbi.nlm.nih.gov). Despite this publication frequency, the awareness of this condition among clinicians is reported to be low and affected patients often suffer a long history of abdominal complaints before they are correctly diagnosed and treated [1]. Therefore, cases reports like this aim at highlighting the condition and at discussing the optimal diagnostic and therapeutical algorithms.

The overall WS incidence is estimated at 0.013–0.3% [1] and can be higher in in burn patients (1.1% of cases) [8] and in patients undergoing scoliosis surgery (up to 2.4% of cases) [9]. Etiological factors leading to WS remain unclear. Although a low BMI, such as present in anorexia, bulimia and other disorders associated with weight loss, may lead to loss of fatty tissue and predispose to WS, additional factors might be decisive for manifestation [1,[10], [11], [12]]. A WS can be suspected in patients with severe eating disorders and with upper gastrointestinal obstruction symptoms, such as postprandial abdominal pain, vomiting, or weight loss [1,13]. However these symptoms are non-specific, making the optimal time-point of diagnostic investigation difficult. Gastrointestinal barium series might be indicative for a WS showing a delay in gastro-duodeno-jejunal transit time of up to 6 h [14]. As barium series do, not reliably exclude a WS, as shown in present case, additional abdominal ultrasound, CT or MRI scan should be performed. Findings of an aorto-mesenteric angle of <25° or an aorto-mesenteric distance of <8 mm define the diagnosis of WS [1,3,3,13]. An additional upper gastrointestinal endoscopy is also necessary to rule out intestinal intraluminal obstruction and other conditions mimicking WS [1,13].

A conservative management is advocated as the first-line therapeutical approach. Propulsive medication might reduce symptoms, but this remains a symptomatic, not curative approach, and might fail to obtain long-term patients satisfaction, as seen in our patient. Nasogastric tube placement for duodenal and gastric decompression and dietary measures to increase body weight, such as use of hypercaloric liquid food, promote restoration of the retroperitoneal fatty tissue with possible consecutive increase of the aorto-mesenteric angle [1,13]. A conservative treatment might be successful in 85% of overall cases, but it is also reported to work best in patients with symptoms lasting for less than a month [15]. However, except patients following scoliosis surgery and burn patients, where the symptoms mostly occur within the first two weeks, the majority of other patients with a WS present with symptoms lasting months to years [1]. The indication for surgery is given in patients not adequately responding to conservative therapy and the duodeno-jejunostomy is the techniques of choice with a reported favorable outcome in 80–90% [1,16].

From the personal perspective, our patient is postoperatively highly satisfied, but clearly advocates that the awareness of a WS as a differential diagnoses of her long-term symptoms should be intensified and therefore clearly supports the publication of this case report. The present study is limited by the singular case reported and the short term follow-up period. However, it clearly highlights the importance of an early suspicion and targeted clarification of a WS in patients with the described symptoms to reduce the high patient’s burden and distinct impairment of quality of life that otherwise may occur in the absence of a correct diagnosis and therapy.

## 4 Conclusion

A low threshold of WS suspicion is necessary to initiate a targeted evaluation and tailored therapy in this rare but impairing condition. A WS condition might be hidden behind presumably more evident diagnoses such as bulimia, significant axial hernia and gastro-esophageal reflux disease in patients with recurred vomiting, abdominal pain and weight loss. Conservative approaches possibly followed by surgical intervention aimed at bypassing the duodenal obstruction lead to a favorable overall outcome in patients with WS.

## Funding

This research did not receive any specific grant from funding agencies in the public, commercial, or not-for-profit sectors.

## Ethical approval

The local ethics committee confirmed that no ethical approval is needed for this case report. Thus, this case report was exempted from requiring an ethical approval by the local ethics committee.

## Consent

Informed consent was obtained from the patient and her parents for the publication of this case report.

## Author’s contribution

All authors have made substantial contributions to all of the following: (1) the conception and design of the study, or acquisition of data, or analysis and interpretation of data, (2) drafting the article or revising it critically for important intellectual content, (3) final approval of the version to be submitted.

## Registration of research Studies

Not applicable.

## Guarantor

Giovanni Frongia.

## Provenance and peer review

Not commissioned, externally peer-reviewed.

## CRediT authorship contribution statement

**Giovanni Frongia:** Conceptualization, Data curation, Formal analysis, Project administration, Writing - original draft, Writing - review & editing. **Jens-Peter Schenk:** Investigation, Validation, Data curation, Writing - review & editing. **Anja Schaible:** Investigation, Validation, Data curation, Writing - review & editing. **Peter Sauer:** Investigation, Validation, Data curation, Writing - review & editing. **Arianeb Mehrabi:** . **Patrick Günther:** Supervision, Validation, Data curation, Writing - review & editing.

## Declaration of Competing Interest

None.
